# Whole-exome sequencing identifies OR2W3 mutation as a cause of autosomal dominant retinitis pigmentosa

**DOI:** 10.1038/srep09236

**Published:** 2015-03-18

**Authors:** Xiangyu Ma, Liping Guan, Wei Wu, Yao Zhang, Wei Zheng, Yu-Tang Gao, Jirong Long, Na Wu, Long Wu, Ying Xiang, Bin Xu, Miaozhong Shen, Yanhua Chen, Yuewen Wang, Ye Yin, Yingrui Li, Haiwei Xu, Xun Xu, Yafei Li

**Affiliations:** 1 Department of Epidemiology, College of Preventive Medicine, Third Military Medical University, Chongqing, People's Republic of China; 2 BGI-Shenzhen, Shenzhen, People's Republic of China; 3 Southwest Hospital/Southwest Eye Hospital, Third Military Medical University, Chongqing, People's Republic of China; 4 Division of Epidemiology, Department of Medicine, Vanderbilt Epidemiology Center, Vanderbilt-Ingram Cancer Center, Vanderbilt University School of Medicine, Nashville, TN, USA; 5 Department of Epidemiology, Shanghai Cancer Institute, Shanghai, People's Republic of China; 6 BGI-Tech, Shenzhen, People's Republic of China

## Abstract

Retinitis pigmentosa (RP), a heterogeneous group of inherited ocular diseases, is a genetic condition that causes retinal degeneration and eventual vision loss. Though some genes have been identified to be associated with RP, still a large part of the clinical cases could not be explained. Here we reported a four-generation Chinese family with RP, during which 6 from 9 members of the second generation affected the disease. To identify the genetic defect in this family, whole-exome sequencing together with validation analysis by Sanger sequencing were performed to find possible pathogenic mutations. After a pipeline of database filtering, including public databases and in-house databases, a novel missense mutation, c. 424 C > T transition (p.R142W) in *OR2W3* gene, was identified as a potentially causative mutation for autosomal dominant RP. The mutation co-segregated with the disease phenotype over four generations. This mutation was validated in another independent three-generation family. RT-PCR analysis also identified that OR2W3 gene was expressed in HESC-RPE cell line. The results will not only enhance our current understanding of the genetic basis of RP, but also provide helpful clues for designing future studies to further investigate genetic factors for familial RP.

Retinitis pigmentosa (RP, MIM#268000), a heterogeneous group of inherited ocular diseases, results in 1 from 3,000 to 5,000 people affecting progressive retinal degeneration^1^. It is clinically characterized by some degenerative symptoms including progressive night blindness, tunnel vision and bone-spicule pigmentation in retina, then cause severe vision impairment and often blindness^2^. The disease is of highly clinical and genetic heterogeneity and could be inherited in autosomal dominant (about 30–40% of total cases), autosomal recessive (50–60%), and X-linked models (5–15%)^3^. Presently, 57 genes/loci have been identified to be associated with RP (https://sph.uth.tmc.edu/RetNet/home.htm), 32 of which were associated with autosomal recessive RP, 20 with autosomal dominant RP, and 5 with X-linked RP. However, a large part of the clinical cases still could not be explained by these genes.

So far, whole-exome sequencing has become a powerful strategy in the detection of rare causal variants of Mendelian disorders, including RP, because disease-causing mutations usually change an encoded protein^4^,^5^,^6^,^7^,^8^,^9^,^10^,^11^,^12^,^13^,^14^,^15^,^16^,^17^. However, many of these studies focus only on simplex families or one affected child from multiplex families. In this study, we selected a large four-generation family, in which six out of nine members in the second generation were affected by RP. Prescreen has excluded known causing mutations for RP. We aimed to identify possible causal genes of RP in this Chinese family using a whole-exome sequencing approach, together with validation by another independent three-generation family.

## Methods

### Subjects and clinical evaluation

**Figure 1:**
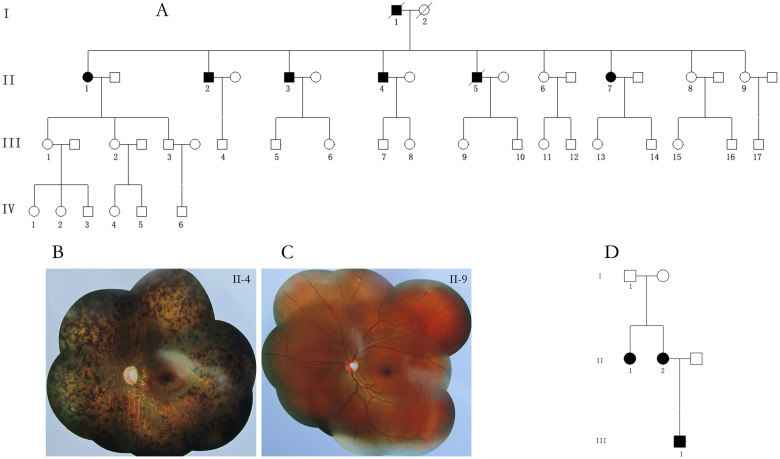
Pedigree of the two familys and fundus photography of an affected case and a normal subject. (A) Pedigree of the four-generation Chinese family with RP: Squares represent males, and circles represent females. Solid symbols indicate affected individuals, while open symbols indicate unaffected individuals. Slash indicates the deceased. (B) Fundus photography of an affected case: attenuation of retinal vascular, bone-spicule pigmentation, Chorioretinal degeneration with peripapillary atrophy, waxy-pale discs, and enlarged optic cups; (C) Normal fundus; (D) Pedigree of the second independent family.

We recruited a four-generation Chinese family from Chongqing in Southwest China. Six of nine members in the second generation affected RP (Figure 1-A). All participants underwent a full ophthalmologic examinations, including slit-lamp biomicroscopy, fundus examination, visual field test, and full-field flash electroretinography (ERG). Blood-derived DNA was available from five cases II-1, II-2, II-3, II-4, II-7 and from twelve healthy family members including II-8, II-9, III-1, III-2, III-3, III-15, III-16, IV-1, IV-2, IV-4, IV-5, IV-6. The study was approved by Ethics Review Committee of Third Military Medical University and carried out in accordance with the Declaration of Helsinki. Peripheral venous blood samples were derived after a signed informed consent.

### DNA extraction, mutation screening

Genomic DNA was extracted from peripheral leukocytes using the TIANamp Blood DNA Kit (Tiangen Biotech Co. Ltd, Beijing, China). To identify whether RP patients in this family were caused by unknown genes, the previously known genes (Supplementary Table 1) shown to be mutated in RP patients were first screened among one RP case (II-1) and one healthy control (II-8) using a targeted gene capture chip developed by BGI, Shenzhen, China. Sanger sequencing was then used to replicate the positive findings.

### Whole-exome sequencing

The whole-exome sequencing approach was employed to identify the disease-associated genes in five subjects, including four RP cases (II-2, II-3, II-4, and II-7) and one healthy control (II-9) by BGI, Shenzhen, China. Thirty microgram (μg) human genomic DNA was extracted from peripheral venous blood samples of each participant. Qualified genomic DNA sample was randomly fragmented by Covaris Acoustic System. Then adapters were ligated to both ends of the resulting fragments. Extracted DNA was then amplified by ligation-mediated PCR (LM-PCR), purified, and hybridized to the Nimblegen SeqCap EZ Library v3.0 (Roche/NimbleGen, Madison, WI) for enrichment. Both non-captured and captured LM-PCR products were subjected to quantitative PCR to estimate the magnitude of enrichment. Each captured library was then loaded on Hiseq2500 platform (Illumina, San Diego, CA). We performed high-throughput sequencing for each captured library to ensure that each sample meets the desired average sequencing depth (90×). Raw image files were processed by Illumina base calling Software 1.7 for base-calling with default parameters and the sequences of each individual were generated as 90 bp pair-end reads.

### Bioinformatics analysis

The clean reads were aligned to the human reference genome (GRCh37, UCSC hg19) by SOAPaligner (soap2.21)^18^. Based on the results from SOAPaligner, software SOAPsnp (version 1.03) was used to assemble the consensus sequence and call genotypes in target regions^19^. When analyzing indel, BWA was used to map reads onto the reference 20, then we passed the alignment result to the Genome Analysis Toolkit (GATK) to identify the breakpoints^21^. Only mapped reads were used for subsequent analysis. Coverage and depth calculations were based on all mapped reads and the exome region. All variants were first filtered against several public databases for the minor allele frequency (MAF) > 0.5%, including dbSNP135, 1000 genomes data (pilot1, 2, 3), hapmap (release 24), YH project^22^, then against two in-house databases (sample size were 7,000 from Vanderbilt Epidemiology Center and 1,414 from BGI, respectively; samples of both databases come from Chinese population, which have the similar genetic background with the subjects in current study).

### Mutation validation

To determine whether any of the remaining variants co-segregated with the disease phenotype in this family, the mutations were then confirmed in all other family members that DNA samples were available by Sanger sequencing. Direct polymerase chain reaction (PCR) products were sequenced using ABI 3730 Genetic Analyzer. Sequencing data were compared pair-wisely with the Human Genome database (GRCh37, UCSC hg19) to detect mutations. The possible causative mutation was further confirmed using RP pedigree database of GBI.

### Cell culture, differentiation and identification

The HESC line H1 was induced to differentiate into retinal pigmented epithelium cells (HESC-RPE) as described previously^23^. Immunofluorescence analysis was performed according previous methods^24^. In brief, the HESC-RPE cells were fixed with 4% paraformaldehyde for 20 min, permeabilized using 0.1% Triton X-100 in PBS for 15 min and blocked for 30 min in 3% BSA. The following primary antibody were used: Mitf (Abcam, 1:50), Pax6 (Abcam, 1:50), zonula occludens-1 (ZO-1, Invitrogen, 1:400).

### Reverse transcription-polymerase chain reaction (RT-PCR)

Total RNA of HESC-RPE on 60 day, 80 d and 100 d were extracted using an RNAprep Pure Cell Kit (Sangon Biotech, CHN) according to the manufacturer's instructions. Total RNA (approximately 1–2 μg per 20 μl reaction) was reverse transcribed using a PrimeScript® RT Reagent Kit (Takara, JPN). PCR amplification of OR2W3 gene (primers: F- TGGTGTTTATCCTGCTCTCTTAC; R- CTCTGTTTCTGAGGGTGTAGATG) was performed by the CFX96 Real-Time PCR System (Bio-Rad, USA) using a PCR Mix (Dongsheng Biotech, CHN) according to the manufacturer's instructions.

## Results

### Clinical characteristics

Figure 1-A presents the pedigree of the four-generation Chinese family, which was consistent with autosomal dominant inheritance. Totally there are **7** members in this family affected RP, including two deceased members (I-1 and II-5). The two deceased members showed similar clinical symptoms and pathogenesis with other 5 alive members (II-1, II-2, II-3, II-4, II-7). Night blindness appeared first, followed by progressive reduction of the visual field, and finally complete blindness in later life. Table 1 presents the clinical data of 5 alive affected individuals. All patients had a progressive bilateral decrease of visual acuity, peripheral visual field, and photophobia. Fundus photography revealed similar clinical features for the affected individuals, including attenuation of retinal vascular, bone-spicule pigmentation, chorioretinal degeneration with peripapillary atrophy, optic disc pallor, and enlarged optic cups, comparing with the normal subject (Figure 1-B, 1-C). ERG records showed no detectable cone or rod responses in the patients.

### Mutation screening

To find the causative mutations and exclude the known genes, we sequenced all exons and the flanking intronic splicing sites of the previously known causative genes of RP (Supplementary Table 1) among one RP case (II-1) and one healthy control (II-8), and confirmed by Sanger sequencing. All genes showed no pathogenic mutations, indicating the possibility of the familial cases in current study were caused by mutations in unknown genes.

### Whole-exome sequencing

Whole-exome sequencing was performed upon five subjects, including four RP cases (II-2, II-3, II-4, and II-7) and one healthy control (II-9). An average of 11,747 MB raw data was generated with a mean depth of 101.74-fold for the target regions. Approximately 98.64% of the targeted bases (64,482,551 bp in length) were covered sufficiently to pass our thresholds for calling SNPs and indels. We identified 144,701-150,367 SNPs and 15368-16173 indels for the five sequenced subjects. For rare inherited diseases, the frequency of the possible pathogenic mutations in healthy population should be very low. Therefore, as shown in Table 2 and Table 3, the results were then filtered against several public variation databases, removing all previously reported variants. We focused only on non-synonymous (NS) variants, variants in splicing sites, and short, frame-shift coding insertions or deletions (Indels). After filtering against these databases, we found 72 SNPs and 15 indels were shared by affected patients and absent in healthy controls. Furthermore, two in-house databases were used to filter the remaining variants, which resulted that 10 SNPs were left (*OR2W3* R142W, *DNM2* R297H, *ROBO2* P1106S, *CSMD3* K3075Q, *ZHX2* G799R, *PALM3* E658Q, *HAP1* E269Q, *BRIP1* N775S, *INTS2* I775L, and *TSSC4* H81R).

### Phenotype & genotype co-segregation and validation of the mutations

**Figure 2:**
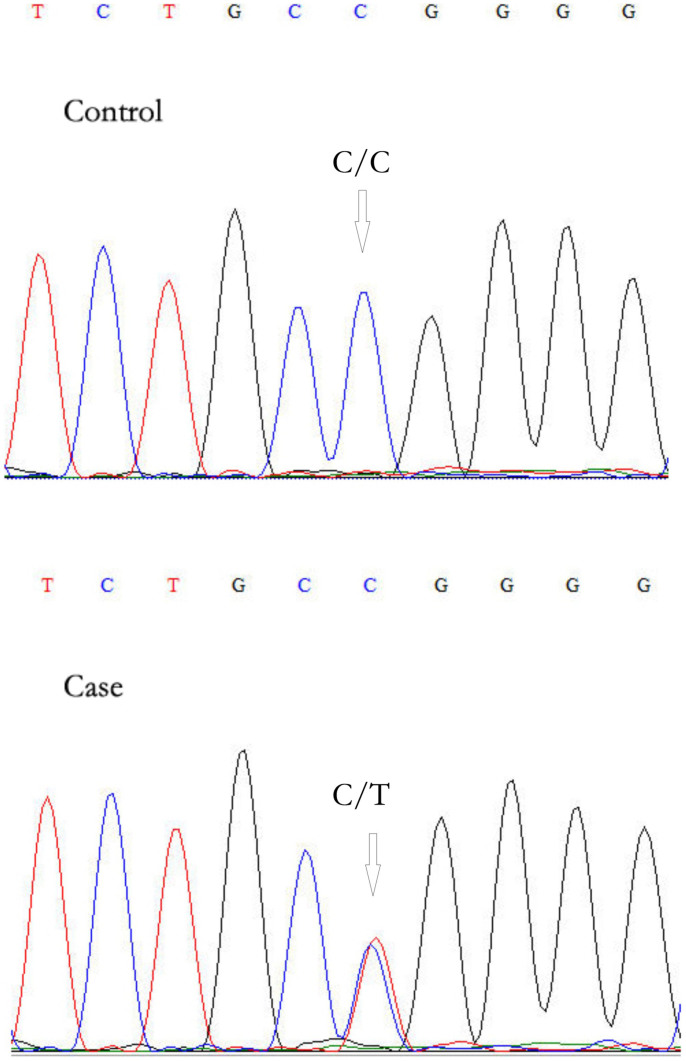
Sanger sequencing of *OR2W3* R142W mutation.

**Figure 3:**
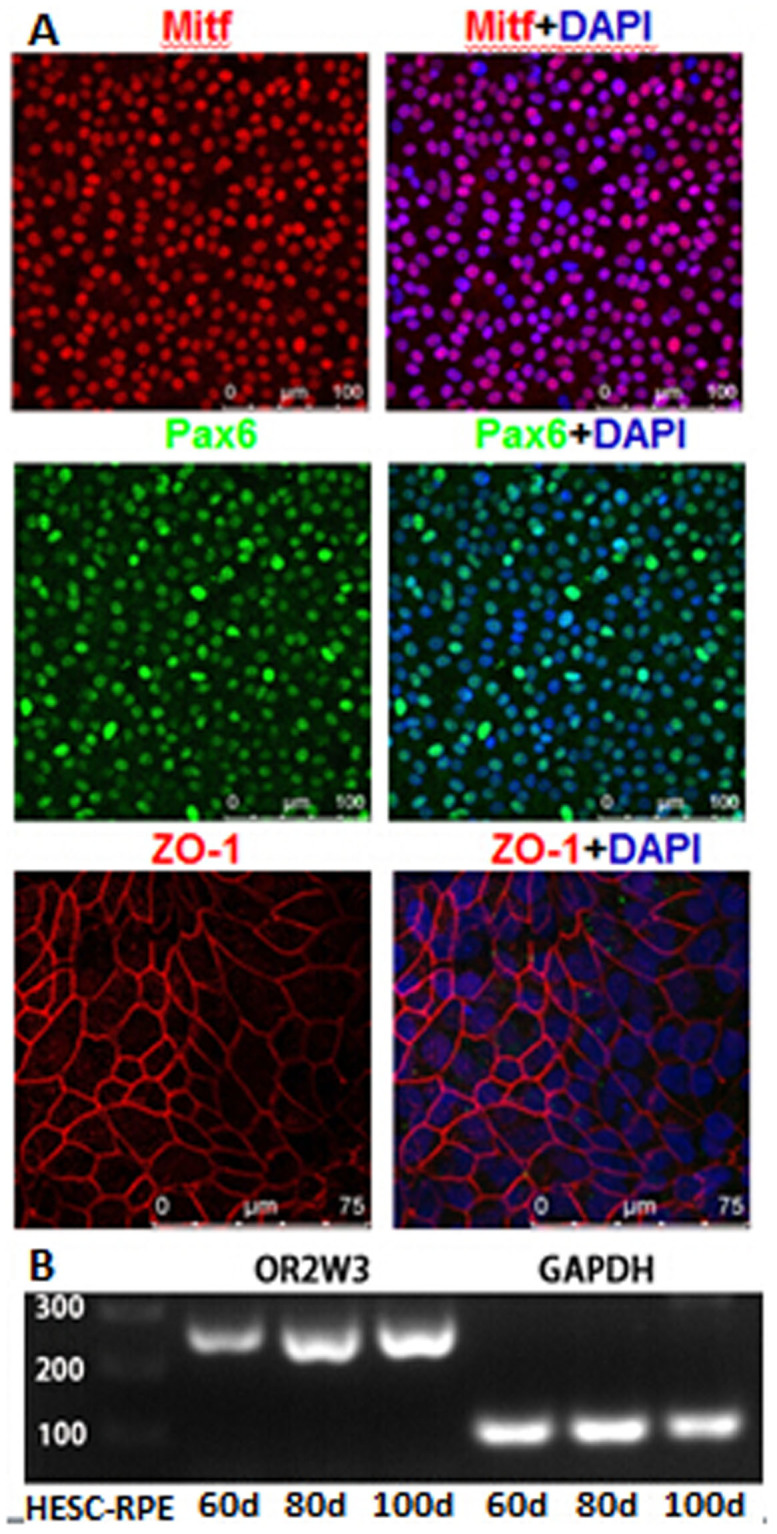
Identification of HESC-RPE cells. (A) Immunocytochemistry of HESC-RPE cells demonstrating the expression of Mitf, Pax6 and ZO-1. (B) RT-PCR analysis of OR2W3 in HESC-RPE. Cropped gel has been run under the same experimental conditions. Full-length blot is presented in Supplementary Figure S1.

The ten remaining mutations were then confirmed in other twelve family members that DNA samples were available by Sanger sequencing to co-segregate with the disease phenotype (Figure 2). Genetic analysis demonstrated that only *OR2W3* (Olfactory receptor 2, W3) R142W was carried by affected patients and absent in healthy controls. Then, *OR2W3* R142W mutation was also observed in another three-generation RP family (Figure 1-D), including 3 cases (II-1, II-2, III-1) and 1 control (I-1); three RP cases were found to carry the same mutation and one healthy control does not. Furthermore, immunofluorescent analysis of HESC-RPE revealed the expression of RPE cells markers (Mitf, PAX6, and ZO-1), while RT-PCR analysis showed that HESC-RPE expressed OR2W3 (Figure 3).

### Conservation of R142W in *OR2W3* gene

Pathogenicity assessment of *OR2W3* R142W mutation was undertaken by evaluation of amino acid evolutionary conservation and in-silico prediction studies. Using UCSC Genome Browser (http://genome.ucsc.edu/cgi-bin/hgGateway), we found the variant was highly conserved in nine primate species, including human, chimp, gorilla, orangutan, bibbon, rhesus, ab-eating_macaque, baboon, green_monkey, and bushbaby, although not conserved in non-primate mammals. According to two web-based topology prediction package: TMPred (http://www.ch.embnet.org/software/TMPRED_form.html) and TopPred (http://mobyle.pasteur.fr/cgi-bin/portal.py?#forms::toppred)^25^, *OR2W3* R142W mutation is located in a transmembrane domain of *OR2W3* gene. The variants was also predicted to have a deleterious effect by Mutation Taster^26^. Exome Variant Server (EVS) database retrieval didn't find this variant.

## Discussion

RP, the most frequent inherited retinal degeneration, has become one of the commonest causes of genetic visual dysfunction^27^. Since RP1 identified by linkage study in 1991^28^, 56 susceptibility genes/loci for RP have been subsequently discovered by different approaches. However, due to the enormous heterogeneity of the disease pathogenesis, a large part of the familial cases still could not be explained. In this study, using a whole exome sequencing approach, we identified a novel missense mutation, c. 424 C > T transition (p.R142W) in *OR2W3* gene, associated with autosomal dominant RP in a large Chinese family. This mutation was validated in another independent three-generation family. RT-PCR analysis also identified that OR2W3 gene was expressed in HESC-RPE cell line. To the best of our knowledge, *OR2W3* gene was identified to be associated with RP for the first time.

The olfactory receptors (ORs), including *OR2W3*, were first defined as a supergene family that encodes G-protein coupled receptor proteins (GPCRs) in olfactory epithelium of the rat in 1991^29^,^30^. Zhao et al. explored the physiological function of ORs in initiating transduction in olfactory receptor neurons^31^. However, ORs were not exclusively expressed in the olfactory epithelium. Recent studies have demonstrated ORs were expressed in a broad variety of other tissues, including autonomic nervous system, brain, tongue, erythroid cells, prostate, placenta, gut and kidney^32^. Furthermore, RNA sequencing of 16 different human tissues by Next Generation Sequencing (NGS) revealed *OR2W3* gene were expressed in 9 different tissue samples, and most highly expressed in thyroid^33^. These indicated the different potential functions of *OR2W3* gene in different human biological process.

*OR2W3* gene, which was located in 1q44, has an intron-free reading frame of 942 nucleotides that encodes 314 amino acids. UCSC Genome Browser^34^ showed that *OR2W3* shares exons with Trim58 (Tripartite motif-containing protein 58). When we used SWISS-MODEL server^35^ to model the structure of OR2W3 protein, JAGGED-1 (PDB ID: 2vj2B)^36^, which was also associated with one kind of autosomal dominant inherited disease - Alagille syndrome^37^, showed the biggest sequence identity with *OR2W3*. Recent studies also revealed that the biological functions of *OR2W3* gene was not only restricted to olfactory system, like G-protein coupled receptor activity and olfactory receptor activity. Aston et al.^38^ and Plaseski et al.^39^ found *OR2W3* rs11204546 was associated with both azoospermia and oligozoospermia risk; a mutation in *OR2W3* gene (chr1:248059606, p.T240P) was associated with the metastasis of pancreatic ductal adenocarcinoma^40^; expression of *OR2W3* was also identified to be associated with long-tern schizophrenia^41^, variability in response tob-blockers^42^, and the changes in global gene-expression profiles in human cervical cancer HeLa cells exposed to non-activated Dendrimers and Dendriplexes^43^. However, through epidemiological survey and Medical record retrieval, all the subjests in current study don't have related diseases and mutations.

Vision and olfaction are two of the major sensory systems, which coordinate and integrate the information to provide us a unified perception of our environment. Studies showed that they share many links and common points in different aspects, including neuroanatomical pathways^44^, cross-modal links and the extension of this notion to goal-directed actions^45^, pathogenic or biological genes^46^,^47^,^48^. Woodard et al.^47^ found *rdgB* (retinal degeneration B), a gene required for normal visual system physiology, was shown to be necessary for olfactory response of both adult flies and larvae, indicating that *rdgB* was required for both visual and olfactory physiology. Loss of olfactory receptor genes were also found to coincide with the acquisition of full trichromatic vision^46^. In this study, we revealed a novel missense mutation in *OR2W3* gene, was associated with autosomal dominant RP. This finding may indicate the essential links between Vision and olfaction, and strongly suggested an exchange in the importance of these two senses.

As we mentioned above, RP refers to a highly clinical and genetic heterogeneous group of inherited ocular diseases. Inheritance patterns included autosomal dominant, autosomal recessive, and X-linked models. In this study, we presumed autosomal dominant to be the inheritance pattern of this family basing on two reasons. First, both the first two generations have affected patients. We excluded the possibility of intermarriage through intensive epidemiologic survey. Second, high prevalence rate (6/9 = 66.7%) in the second generation. Nevertheless, we also analyzed the data based on the autosomal recessive model, including homozygous inheritance model and compound heterozygous model, but no promising mutations were detected. One limitations of this study is that due to patient's refusal for retinal biopsy, the results could not be strengthened by RNA analysis of this gene or immune-localisation of the protein using multiple tissues including the retina and retinal pigment epithelium(RPE) cells.

## Conclusion

A novel missense mutation (*OR2W3* R142W) was identified to be associated with RP by whole-exome sequencing. Our findings expand the phenotypic and mutation spectrum of RP and provide helpful clues for designing future studies to further investigate genetic factors for familial RP.

## Author Contributions

L.Y., X.X., X.H., M.X. and G.L. designed the experiments; M.X., G.L., W.W., Z.Y., W.N., W.L., X.Y. and X.B. performed the investigations and experiments; M.X. and G.L. analyzed the data; L.L., Z.W., G.Y., L.J., S.M., C.Y., W.Y., Y.Y. and L.Y. provided technical and material support. M.X. and G.L. wrote the manuscript; all authors reviewed the manuscript.

## Supplementary Material

Supplementary Information Supplementary Information

## Figures and Tables

**Table 1:** Characteristics of 5 alive affected individuals from RP pedigree

Characteristics	II-1	II-2	II-3	II-4	II-7
Age (years)	64	60	58	54	46
Gender	Female	Male	Male	Male	Female
Age of night blindness onset (years)	20	30	21	20	30
Visual field	None	None	None	None	None
Optic disc	pallor	pallor	pallor	pallor	pallor
Artery attenuation	Yes	Yes	Yes	Yes	Yes
Pigment deposits	Yes	Yes	Yes	Yes	Yes
Electroretinography	non-detectable	non-detectable	non-detectable	non-detectable	non-detectable

**Table 2:** Number of candidate variants filtered against several public variation databases

Feature_SNP	control:II-9	case:II-2	case:II-3	case:II-4	case:II-7
Total_SNPs^1^	150367	146587	149036	144701	147040
Functional_SNPs^2^	15982	15944	15897	15814	15847
Filtered_DBsnp	13286	13229	13202	13084	13195
Filtered_DBsnp_1000gene	2857	2834	2804	2716	2709
Filtered_DBsnp_1000gene_Hapmap	2817	2793	2763	2678	2670
Filtered_DBsnp_1000gene_Hapmap_YH	2664	2638	2609	2524	2515
Filtered_DBsnp_1000gene_Hapmap_YH_II-9^3^	0	870	840	786	850
Share_all_cases	72				
Filtered _Housedatabase	10				
Genotype & phenotype coseparation	1 (*OR2W3* R142W)				

^1^Total-SNPs detection were performed on the targeted exome regions and flanking regions within 200 bp. SNP types include variants of nonsense, missense, splicing site, 5-UTR, 3-UTR, NR_exon, synonymous-coding, intron, intergenic.

^2^Functional_SNPs include variants of nonsense, missense, splicing site.

^3^In this step, variants were filtered by mutations of healthy control: II-9.

**Table 3:** Number of candidate Indels filtered against several public variation databases

Feature_Indel	control:II-9	case:II-2	case:II-3	case:II-4	case:II-7
Total_Indels^1^	16173	15403	16053	15368	15772
Functional_Indels^2^	2053	1976	2058	1996	2089
Filtered_DBsnp	586	576	582	570	565
Filtered_DBsnp_1000gene	337	346	323	325	327
Filtered_DBsnp_1000gene_Hapmap	337	346	323	325	327
Filtered_DBsnp_1000gene_Hapmap_YH	335	344	321	324	325
Filtered_DBsnp_1000gene_Hapmap_YH_ II-9^3^	0	159	147	146	151
Share_all_cases	15				
Housedatabase_filter	0				

^1^Total-Indels detection were performed on the targeted exome regions and flanking regions within 100 bp. Indel types include variants of frameshift, cds-Indel, spliceSite, 5-UTR, 3-UTR, intron, promoter, intergenic.

^2^Functional_Indels include variants of frameshift, cds-Indel, spliceSite.

^3^In this step, variants were filtered by mutations of healthy control: II-9.
