# Supplementary material for: Whole-exome sequencing identifies OR2W3 mutation as a cause of autosomal dominant retinitis pigmentosa
Source: Sci Rep. 2015 Mar 18;5:9236. doi: 10.1038/srep09236 (PMC4363838; doi:10.1038/srep09236)
Supplement: Supplementary Information [file srep09236-s1.doc]

**Whole-Exome Sequencing Identifies OR2W3 Mutation as a Cause of Autosomal Dominant Retinitis Pigmentosa**

**Running title: OR2W3 Mutation and Retinitis Pigmentosa**

Xiangyu Ma ^1 *^, Liping Guan ^2 *^, Wei Wu ^3^, Yao Zhang^1^, Wei Zheng ^4^, Yu-Tang Gao ^5^, Jirong Long ^4^, Na Wu ^1^, Long Wu ^1^, Ying Xiang ^1^, Bin Xu ^1^, Miaozhong Shen ^2^, Yanhua Chen ^2^, Yuewen Wang ^2^, Ye Yin ^2^, Yingrui Li ^2,6^, Haiwei Xu ^3^, Xun Xu^2 #^, Yafei Li ^1 #^

^1^ Department of Epidemiology, College of Preventive Medicine, Third Military Medical University, Chongqing, People’s Republic of China

^2^ BGI-Shenzhen, Shenzhen, People’s Republic of China

3 Southwest Hospital/Southwest Eye Hospital, Third Military Medical University, Chongqing, People's Republic of China

^4^ Division of Epidemiology, Department of Medicine, Vanderbilt Epidemiology Center, Vanderbilt-Ingram Cancer Center, Vanderbilt University School of Medicine, Nashville, TN, USA

^5^ Department of Epidemiology, Shanghai Cancer Institute, Shanghai, People’s Republic of China

^6^ BGI-Tech, Shenzhen, People’s Republic of China；

^*^ These authors contributed equally to this work.

^#^ These authors jointly directed the project.

**Correspondence:**

Yafei Li, Ph.D.

Department of Epidemiology, College of Preventive Medicine, Third Military Medical University, Chongqing 400038, People’s Republic of China

E-mail: liyafei2008@hotmail.com

Telephone: +86 23 68752293

Fax: +86 23 687522893

| **Supplementary Table 1. List of genes previously shown to be mutated in RP patients** |  |
| --- | --- |
| **Disease** | **Gene** |
| Retinitis pigmentosa 1 | *RP1* |
| Retinitis pigmentosa 2 | *RP2* |
| Retinitis pigmentosa 3，X-linked | *RPGR* |
| Retinitis pigmentosa 4 | *RHO* |
| Retinitis pigmentosa 7 | *PRPH2* |
| Retinitis pigmentosa 7, digenic | *ROM1/PRPH2* |
| Retinitis pigmentosa 9 | *RP9* |
| Retinitis pigmentosa 10 | *IMPDH1* |
| Retinitis pigmentosa 11 | *PRPF31* |
| Retinitis pigmentosa 12 | *CRB1* |
| Retinitis pigmentosa 13 | *PRPF8* |
| Retinitis pigmentosa 14 | *TULP1* |
| Retinitis pigmentosa 17 | *CA4* |
| Retinitis pigmentosa 18 | *PRPF3* |
| Retinitis pigmentosa 19 | *ABCA4* |
| Retinitis pigmentosa 20 | *RPE65* |
| Retinitis pigmentosa 25 | *EYS* |
| Retinitis pigmentosa 26 | *CERKL* |
| Retinitis pigmentosa 27 | *NRL* |
| Retinitis pigmentosa 28 | *FAM161A* |
| Retinitis pigmentosa 30 | *FSCN2* |
| Retinitis pigmentosa 31 | *TOPORS* |
| Retinitis pigmentosa 33 | *SNRNP200* |
| Retinitis pigmentosa 35 | *SEMA4A* |
| Retinitis Pigmentosa 36 | *PRCD* |
| Retinitis pigmentosa 37 | *NR2E3* |
| Retinitis pigmentosa 38 | *MERTK* |
| Retinitis pigmentosa 39 | *USH2A* |
| Retinitis pigmentosa 40 | *PDE6B* |
| Retinitis Pigmentosa 41 | *PROM1* |
| Retinitis pigmentosa 42 | *KLHL7* |
| Retinitis pigmentosa 43 | *PDE6A* |
| Retinitis pigmentosa 44 | *RGR* |
| Retinitis pigmentosa 45 | *CNGB1* |
| Retinitis pigmentosa 46 | *IDH3B* |
| Retinitis pigmentosa 47 | *SAG* |
| Retinitis pigmentosa 48 | *GUCA1B* |
| Retinitis pigmentosa 49 | *CNGA1* |
| Retinitis pigmentosa 50 | *BEST1* |
| Retinitis pigmentosa 51 | *TTC8* |
| Retinitis pigmentosa 53 | *RDH12* |
| Retinitis pigmentosa 54 | *C2orf71* |
| Retinitis pigmentosa 55 | *ARL6* |
| Retinitis pigmentosa 56 | *IMPG2* |
| Retinitis pigmentosa 57 | *PDE6G* |
| Retinitis pigmentosa 58 | *ZNF513* |
| Retinitis pigmentosa 59 | *DHDDS* |
| Retinitis pigmentosa 60 | *PRPF6* |
| Retinitis pigmentosa 61 | *CLRN1* |
| REtinitis pigmentosa 62 | *MAK* |
| Retinitis pigmentosa 65 | *CDHR1* |
| Retinitis pigmentosa | *RBP3* |
| X-linked Retinitis pigmentosa, and sinorespiratory infections, with or without deafness | *RPGR* |
| recessive retinitis pigmentosa and mental retardation | *CC2D2A* |
| Posterior column ataxia with retinitis pigmentosa | *FLVCR1* |
| *TTPA*-related Retinitis pigmentosa | *TTPA* |
| X-linked retinitis pigmentosa, severe | *OFD1* |
| retinitis pigmentosa autosomal recessive (ARRP) | *RLBP1* |
| Ataxia, posterior column, with retinitis pigmentosa | *FLVCR1* |
| Bietti crystalline corneoretinal dystrophy | *CYP4V2* |
| Retinitis pigmentosa, juvenile | *SPATA7* |
| Retinitis pigmentosa, juvenile | *AIPL1* |
| Retinitis pigmentosa, juvenile | *LRAT* |
| Retinitis pigmentosa, juvenile | *TULP1* |
| Retinitis pigmentosa, concentric | *BEST1* |

**Supplementary Figure S1 Full-length blot of RT-PCR analysis of OR2W3 in HESC-RPE**
